# Double Tract vs. Roux-en-Y Reconstruction in the treatment of Gastric Cancer

**DOI:** 10.12669/pjms.343.14348

**Published:** 2018

**Authors:** Aleksandar Resanovic, Tomislav Randjelovic, Vladimir Resanovic, Borislav Toskovic

**Affiliations:** 1 Dr. Aleksandar Resanovic, Department of Surgery, University Hospital Bezanijska Kosa, Belgrade, Serbia; 2 Prof. Tomislav Randjelovic, Medical Faculty, University of Belgrade, Belgrade, Serbia. Department of Surgery, University Hospital Bezanijska Kosa, Belgrade, Serbia; 3 Dr. Vladimir Resanovic, Emergency Center, Clinic for Urgent Surgery, Clinical Center Of Serbia, Belgrade, Serbia. Department of Surgery, University Hospital Bezanijska Kosa, Belgrade, Serbia; 4 Dr. Borislav Toskovic, Department of Surgery, University Hospital Bezanijska Kosa, Belgrade, Serbia

**Keywords:** BMI, Double tract, Gastric cancer, Roux-en-Y

## Abstract

**Objective:**

Functional outcomes were prospectively compared between the standard Roux-en-Y and Double-tract reconstruction following a total gastrectomy and D2 lymphadenectomy.

**Methods:**

One hundred ten patients with gastric cancer were divided into two groups by the type of reconstruction. Age, gender, T stage, AJCC stage, length of operation, BMI (body mass index, kg/m2), time to soft diet, postoperative leakage of the esophagojejunostomy (EJS), stricture of the EJS, meal intake, and quality of life (QOL) were recorded.

**Results:**

The mean age in the R-Y group was 61.57, with the SD of 9.53, while in the DT group the mean age was 60.17 with a SD of 9.92. The BMI decline in the R-Y group was 4.09 with a SD of 1.11, while in the DT group it was 2.85 with a SD of 1.27. We found a highly significant statistical difference between the two groups in the rate of the BMI decline (p<0,001). We found no statistically significant difference regarding QOL between the two groups, p>0.05.

**Conclusions:**

The Double tract reconstruction is a simple procedure and the rate of the BMI decline is much smaller compared to the Roux-en-Y group.

## INTRODUCTION

Almost one million (951 600) new cases of gastric cancer (GC) were diagnosed globally in 2012, resulting in approximately 723 100 deaths.^1^ It is very important to point out the variation in incidence on a global level. The countries with the highest rates for GC remain the east Asian, the south American and east European countries, while the incidence is significantly lower for the population of Northern America and western European countries.^2^ 140 000 cases and 107 000 deaths occurred in Europe annually.^3^ Over the past 50 years a steady decline in GC incidence has been observed, not only in north America or western Europe, but also in the countries with high prevalence for GC. The extent of resection in GC patients is determined by the preoperative stage. Surgical treatment for gastric cancer, especially at an early stage, can be a curative procedure. For stage IB–III gastric cancer, radical gastrectomy is indicated. To allow reliable staging, an excision of a minimum number of 15 lymph nodes is recommended by the UICC/AJCC TNM (seventh edition) classification.^4^ In Asian countries; experience from observational and randomised trials demonstrates that D2 dissection leads to superior outcomes compared with D1 resection.^5^ Patients in our study were presented with tumors which were T2, T3 and T4a. In these cases we were obligated to perform a standard gastrectomy with D2 lymphadenetomy

The first successful total gastrectomy was performed by George Schlatter in Zurich in 1897, while the second one was performed in the United States by Charles Brooks Brigham in 1898 at St. Luke’s Hospital in San Francisco.^6^ Various methods of reconstruction after TG have been devised over time. However no optimal reconstruction method has yet become universally accepted. The Rouxen-Y anastomosis (R-Y), first applied by Orr after TG^7^, is still utilized as the preferred reconstruction in Japan, as well as in many Western countries, because it is simple toper form and decreases esophageal reflux.^8^ In 1965, Kajitani and Sato reported the use of double tract (DT) reconstruction. With this procedure, an esophagojejunostomy (EJS) is performed as with the R-Y technique, and duodenojejunostomy is added about 20 cm distal from the EJS.^9^ At our hospital, most surgeons prefer to perform a standard R-Y reconstruction with a circular stapler used for the EJS, while the DT reconstruction is becoming more and more a part of standard surgical practice.

In this study functional outcomes were prospectively compared between the standard Roux-en-Y and Double-tract reconstruction following a total gastrectomy and D2 lymphadenectomy for gastric cancer. A comparison of quality of life (QOL) between the two groups was also examined.

## METHODS

Between 2012 and 2016, 110 patients diagnosed with adenocarcinoma of the stomach, age between 35 and 74, were included in this study and operated by three experienced surgeons. Patients who had a malignant peritoneal dissemination or distant metastasis and patients who were unable to cooperate or were in poor general health were excluded from this study. Age, gender, T stage, AJCC stage, comorbidities, length of operation, BMI (body mass index, kg/m2), time to soft diet, postoperative leakage of the esophagojejunostomy (EJS), stricture of the EJS, meal intake, as well as the quality of life (QOL) were recorded. BMI was assessed preoperatively, 12 months after the operation as well as the decline in BMI. Meal intake was evaluated at 3, 6 and 12 months and was described in percentage change in reference to the level of preoperative meal intake. Postoperative leakage at the level of EJS was determined with a gastrografin contrast study, while stricture of the EJS was outlined after 12 months on an upper GI endoscopy, and was treated with a ballon dilatation. The QOL was assessed using a QLQ-C30 questionnaire established by the European Organisation for Research and Treatment of Cancer (EORTC). The overall QOL was distributed on a scale from one to seven, with one being a very poor QOL, and 7 being an excellent QOL.

The aim of the study was to compare two surgical procedures different in reference to the type of reconstruction used (Roux-en-Y vs. Double tract reconstruction) after total gastrectomy, D2 lymphadenectomy and omentectomy performed for gastric cancer. The type of reconstruction was chosen randomly using the sealed envelope system. This study was designed as a stratified randomized trial with two stratums (groups) according to the type of reconstruction that the patients were submitted to.

The R-Y reconstruction is characterized (after resection) by an EJS of the distal esophagus to a jejunal limb (mostly the second jejunal loop), which has been excluded from the normal intestinal passage. The EJS was created as a retrocolic, end-to-side anastomosis with a circular stapler, while the blind closure of the proximal duodenum was performed with a linear stapler. Afterwards, a second two-layer manual anastomosis, between the ascended jejunal limb and the first jejunal loop that carries the bilio-pancreatic juice, was created. Food passes through the esophagus to the jejunal loop, and mixes with bile and pancreatic juice 40 cm below. The distance between the enteroenterostomy and EJS (about 40 cm) minimizes biliary content and pancreatic juice reflux to the esophagus.

The DT reconstruction is characterized (after resection) by a jejuno-duodenostomy of the duodenum to a jejunal limb (mostly the second jejunal loop), which has already been used for the creation of a proximal EJS. In the double-tract procedure we interposed a 30 cm segment of the jejunum between the esophagus and the duodenum. The second enteroenterostomy is performed 20-25 cm below. Therefore, due to the duodeno-intestinal anastomosis part of the nutritional content passes to the duodenum mixing with the biliary content and pancreatic juice. The digestive and absorption functions of the duodenum are maintained.

### Statistical analysis

Statistical analysis was performed with SPSS 21.0 program. Apart from descriptive statistic methods (mean, standard deviation), we used t-test and chi-squared test for quantitative comparisons. P<0.05 was considered statistically significant, and p<0.001 was considered highly statistically significant.

### Ethics committee

The study was approved by the Ethics Committee of the University Hospital Bezanijska Kosa, written informed consent was obtained from each human subject and the patients had been operated and postoperatively closely monitored in the department of abdominal surgery of the University Hospital Bezanijska Kosa. The procedures followed ethical standards according to the Declaration of Helsinki of 1975, revised in 2013.

## RESULTS

Our study involved a total number of 110 patients, 70 of them were male, 40 of them were female (Table-I). Patients were divided into two groups, with the defining factor being the type of reconstruction. A total number of 51 patients were reconstructed with the R-Y method, while 59 of them reconstructed with the DT method. In the R-Y group, there were 40 male and 11 female patients, while in the DT group; there were 30 male and 29 female patients. Comparing the groups in regards to gender, we found that there was a statistically significant difference p=0.028 (p<0.05).

**Table-I:** Baseline characteristics of a total number of 110 patients divided into two groups.

Variable	Roux-en-group group (n=51)	Double tract group (n=59)	Statistical significance
Age	61.57+/-9.53	60.17+/-9.92	NS
Sex (M/F)	40/11	30/29	NS
Stage AJCC (IIa)	5	15	P<0.05
Stage AJCC (IIb/IIIa/IIIb)	18/17/11	17/20/7	NS
Operation time (minutes)	193.41+/-13.87	216.01+/-12.89	P<0.001
Time to soft diet	6.82+/-2.33	5.73+/-2.13	P<0.05
Preop BMI(kg/m^2^)	25.24+/-1.65	25.39+/-1.36	NS
BMI decline (12 months after surgery)	4.09+/-1.11	2.85+/-1.27	P<0.001
EJS leakage (%) EJS stricture after 12 months (%)	5.9 7.84	5.1 8.47	NS NS

The mean age in the R-Y group was 61.57, with the SD of 9.53, while in the DT group the mean age was 60.17 with a SD of 9.92. Comparing the results, we found no statistically significant difference between the two groups, p<0.05 (p= 0.4539).

We analysed the distribution of patients in the two groups in reference to the AJCC stage. We found a statistically significant difference in the presence of stage IIa gastric cancer between the two groups p=0.035 (p<0.05). The preoperative values of the BMI in the R-Y group was 25.24 with a SD of 1.65, while in the DT group it was 25.39 with a SD of 1.36. On follow-up after 12 months, the BMI value in the R-Y group was 21.14 with a SD of 1.64, while in the DT group it was 22.55 with a SD of 1.58. The BMI decline in the R-Y group was 4.09 with a SD of 1.11, while in the DT group it was 2.85 with a SD of 1.27. We found a highly significant statistical difference between the two groups in the rate of the BMI decline following 12 months from surgery (p<0.001).

The mean time of the soft diet intake in the R-Y group was 6.82, with a SD of 2.33, while in the DT group the mean time was 5.73 with a SD of 2.13. Comparing the two groups, we have found that there was a statistically significant difference between them p<0.05 (0.0115). There was a significant decrease in meal intake three months after surgery. In the R-Y group it was at 61.64% of the preoperative value, while in the DT group it was 65.94%. There was a gradual rise in terms of percentages in both groups after 6 and 12 months, but no statistically significant difference between the two groups was determined (p>0.05). The QOL assessment was made 12 months after surgery using a QLQ-C30 questionnaire. Out of 110 patients included in this study, a fully filled questionnaire was obtained from 104 patients (50 of them were in the R-Y group, while 54 of them were in the DT group). We found no statistically significant difference regarding QOL between the two groups, p>0.05. The mean length of the procedure with the R-Y type of reconstruction was 193.41minutes with a SD of 13.87 minutes. It was shorter than the mean time needed to perform the operation with the DT reconstruction which was 216.01 with a SD of 12.89. Comparing the two groups, we have found that there was a highly significant statistical difference between them (p<0.001).

The total number of leakage at the EJS was six, and they were equally distributed between the two groups, with 5.90% of patients with a fistula in the R-Y group, and 5.10% of patients in the DT group. Comparing the results, we found no statistically significant difference between the two groups p=0.85 (p>0.05). Analyzing the occurrence of stricture of esophagojejunostomy after 12 months, we found a total number of 9 patients with a stricture.

1. 7.84% of patients in the R-Y group
2. 8.47% of patients in the DT group

Comparing the results, we found no statistically significant difference between the two groups p=0.90 (p>0.05).

## DISCUSSION

Less than a century ago, gastric cancer (GC) was the most common cancer in the United States and perhaps throughout the world. Although it is no longer the most common cancer worldwide, GC remains the second leading cause of cancer-related mortality worldwide and the most prevalent cancer in Eastern Asia.^10^ It is considered that worldwide, approximately one million people are annually diagnosed with GC, and that about 750.000 die from this disease annually^11^, which makes GC a cancer with the 4^th^ most common incidence and the 2^nd^ most common cause of cancer related death.^12^ GC also causes one of the highest cancer burdens, as measured by disability-adjusted life year’s lost.^13^

According to EUCAN, the 2012 incidence rates for GC in Serbia per 100.000 were 12.3, while the mortality rate was 9.8.^14^ Originating from a developing country, the ever present problem in gastric cancer treatment in Serbia is outlined by the fact that the majority of the patients that we treat is being diagnosed with gastric cancer after the occurrence of symptoms, such as weight loss, dysphagia, dyspepsia, vomiting, early satiety and/or iron deficiency anemia. The problem lies in the fact that there is no routine screening program present for GC in Serbia.

Especially at an early stage, surgical treatment for gastric cancer can be a curative procedure. For stage IB–III gastric cancer, radical gastrectomy is indicated. To allow reliable staging, an excision of a minimum number of 15 lymph nodes is recommended by the UICC/AJCC TNM (seventh edition) classification. Consensus opinion is that, in Western countries, medically fit patients should undergo D2 dissection that is carried out in specialized, high-volume centers with appropriate surgical expertise and postoperative care [I, B].^15^-^17^ Since all of the patients included in this study have been stage IIA, IIB, IIIA and IIIB, a standard radical gastrectomy with D2 lymphadenectomy was performed.

Acknowledging the fact that the treatment results of patients diagnosed with GC have markedly improved over the years, focusing on the reconstruction type after total gastrectomy is important in terms of a promising QOL. The simpler the construction method is, the better it is in terms of postoperative QOL.^18^ An increase in postoperative complications may be present in cases when the surgical procedure is complicated, and once patients suffer some complications, it compromises the quality of postoperative life. The DT reconstruction is as simple as the R-Y reconstruction, and it can be safely performed even after a total gastrectomy with extended lymphadenectomy.^19^ Digestion and absorption of many substances, such as proteins, fats, fat-soluble vitamins, most water-soluble vitamins (except vitamin B12), and selected microelements (iron, potassium) takes place in the duodenum and initial part of the jejunum. Therefore, the maintenance of partial duodenal passage should in theory improve absorption, even in other segments of the bowel.^20^-^22^

The purpose of this study was to assess the outcomes of the two different types of reconstruction used in patients diagnosed with gastric cancer. We assessed the operative time length, the time needed to start with the soft diet, meal intake, the decline of the BMI after 12 months from surgery, occurrence of EJS leakage and stricture of the EJS after 12 months, as well the QOL using a QLQ-C30 questionnaire. A total number of 110 patients were evaluated, with 51 patients being reconstructed with the R-Y method, while 59 patients were reconstructed with the DT method. We established a mean operation time in the Roux group of 193.41with a SD of 13.87 minutes, while in the DT group it was 216.01 with a SD of 12.89 minutes. We found that there was a highly significant statistical difference between them (p<0.001). Comparing to the results published by Iwahashi et al.^18^, we found that it took us 66.59 minutes less to perform the R-Y procedure, and 37.99 minutes less to perform the DT procedure, while Iwahashi et al.^18^ Found that there was no statistically significant difference between the time needed to do the R-Y and DT reconstruction.

The mean time needed to start with the soft diet in the R-Y group was 6.82 days, with a SD of 2.33, while in the DT group the mean time was 5.73 days with a SD of 2.13. We established a statistically significant difference between the two groups p<0.05 (0.0115). In a paper published by Hur et al.^23^, the mean time to soft diet in the R-Y group was 5.6, while in the group with the inclusion of the duodenum it was 5.5. The results published in this study regarding the meal intake, as well as the QOL assessed by with the QLQ-C30, show no statistically significant difference, and are in aligment with the results published in other studies on GC. Assessing the number of anastomotic leaks, we found a total number of 6 leakages of the EJS in all 110 cases, which were equally distributed between the two groups, with no statistically significant difference between them. Analyzing the data published by Bandurski et al.^24^, we experienced a higher percentage of EJS leak (5.08%) compared to their study (2.6%), but we recorded no cases with a leakage of the enteroenterostomy, nor did we experience any leakage of the duodenojejunostomy. In a paper published by Namikawa et al.^25^ the authors experienced no leakage of the EJS in a total number of 71 patients. Total number of 9 patients with a stricture of the EJS was recorded on follow-up after 12 months from surgery on a routine upper GI endoscopy. No statistically significant difference was recorded between the groups. In comparison to the results published by Fukuhara et al.^26^, we found that we experienced a slightly higher percentage of EJS stricture 8.18% to 7.0%.

Regarding the decline rate of the BMI 12 months after surgery, we found that there was a highly significant statistical difference between the groups (p<0.001). In contrast to the opinion of other authors, we feel that this method of reconstruction could potentially bring advantages to patients in the postoperative period; therefore it is still worth conducting prospective clinical trials comparing the sere constructions in the future.

## CONCLUSIONS

The benefits of the DT reconstruction are:

1. A simple procedure
2. Preservation of the duodenal passage
3. No duodenal stump, resulting in no risk of postoperative stump rupture
4. The 12-month decline of the BMI is smaller compared to the R-Y reconstruction.

### Author`s contribution

**AR** conceived, designed and did statistical analysis & editing of manuscript.

**VR, BT** did data collection and manuscript writing.

**TR** did review and final approval of manuscript.
